# Renal imaging in 199 Dutch patients with Birt-Hogg-Dubé syndrome: Screening compliance and outcome

**DOI:** 10.1371/journal.pone.0212952

**Published:** 2019-03-07

**Authors:** Paul C. Johannesma, Irma van de Beek, Tijmen J. W. T. van der Wel, Rinze Reinhard, Lawrence Rozendaal, Theo M. Starink, Jan Hein T. M. van Waesberghe, Simon Horenblas, Hans J. J. P. Gille, Marianne A. Jonker, Hanne E. J. Meijers-Heijboer, Pieter E. Postmus, Arjan C. Houweling, Jeroen R. A. van Moorselaar

**Affiliations:** 1 Department of Pulmonary Diseases, Amsterdam UMC, Vrije Universiteit Amsterdam, Amsterdam, the Netherlands; 2 Department of Surgery, Utrecht University Medical Center, Utrecht, the Netherlands; 3 Department of Clinical Genetics, Amsterdam UMC, Vrije Universiteit Amsterdam, Amsterdam, the Netherlands; 4 Department of Radiology, OLVG, Amsterdam, the Netherlands; 5 Department of Pathology, Amsterdam UMC, Vrije Universiteit Amsterdam, Amsterdam, the Netherlands; 6 Department of Dermatology, Leiden University Medical Center, Leiden, the Netherlands; 7 Department of Radiology, Amsterdam UMC, Vrije Universiteit Amsterdam, Amsterdam, the Netherlands; 8 Department of Urology, the Netherlands Cancer Institute, Amsterdam, the Netherlands; 9 Department of Epidemiology and Biostatistics, Amsterdam, the Netherlands; 10 Department for Health Evidence, Radboud University Medical Center, Nijmegen, the Netherlands; 11 Department of Pulmonology, Leiden University Medical Center, Leiden, the Netherlands; 12 Department of Urology, Amsterdam UMC, Vrije Universiteit Amsterdam, Amsterdam, the Netherlands; National Institute of Health, UNITED STATES

## Abstract

Birt-Hogg-Dubé syndrome is associated with an increased risk for renal cell carcinoma. Surveillance is recommended, but the optimal imaging method and screening interval remain to be defined. The main aim of our study was to evaluate the outcomes of RCC surveillance to get insight in the safety of annual US in these patients. Surveillance data and medical records of 199 patients with Birt-Hogg-Dubé syndrome were collected retrospectively using medical files and a questionnaire. These patients were diagnosed in two Dutch hospitals and data were collected until June 2014. A first screening for renal cell carcinoma was performed in 172/199 patients (86%). Follow-up data were available from 121 patients. The mean follow-up period per patient was 4.2 years. Of the patients known to be under surveillance, 83% was screened at least annually and 94% at least every two years. Thirty-eight renal cell carcinomas had occurred in 23 patients. The mean age at diagnosis of the first tumour was 51. Eighteen tumours were visualized by ultrasound. Nine small tumours (7–27 mm) were visible on MRI or CT and not detected using ultrasound. Our data indicate that compliance to renal screening is relatively high. Furthermore, ultrasound might be a sensitive, cheap and widely available alternative for MRI or part of the MRIs for detecting clinically relevant renal tumours in BHD patients,but the limitations should be considered carefully. Data from larger cohorts are necessary to confirm these observations.

## Introduction

Birt-Hogg-Dubé syndrome (BHD, OMIM #315150) is an autosomal dominant condition caused by germline mutations in the *FLCN* gene encoding folliculin and is characterized by fibrofolliculomas, lung cysts, spontaneous pneumothorax and renal cell carcinoma (RCC) [1]. Around 3–5% of all RCCs is estimated to have a hereditary cause [2]. Hereditary RCCs differ from the far more common sporadic form in several aspects. Hereditary tumours often present at a relatively younger age, are often multifocal and/ or bilateral and may have a characteristic histology. In addition, they may be associated with recognizable syndromic features besides RCC. The family history may be positive for RCC or for associated syndromic clinical features. In patients with BHD, the prevalence of RCC is estimated to be 16–34% with a mean age at diagnosis of 50 years [3–6]. The most commonly reported histological subtypes of BHD associated renal tumours is hybrid oncocytic/chromophobe renal cell carcinoma. However, other subtypes also occur [3, 5–10]. Renal surveillance in BHD patients has been recommended from age 20, preferably by annual MRI (Magnetic Resonance Imaging) [11]. This is based on the high sensitivity and the lack of radiation exposure of MRI. However, its availability and costs may be limitations in clinical practice. To our knowledge, evaluation of renal screening in BHD patients has not been previously performed. To gain insight in the optimal screening regimens in rare disorders, it is crucial to study available data of patient cohorts. The recommended screening program in the Dutch hospitals has changed over the years based on expert opinion. A first screening in BHD patients has mostly consisted of both MRI and ultrasound (US). All patients were advised to perform annual follow-up. Dependent on the moment in time and the hospital where the diagnosis was established, the follow-up consisted of MRI, US, CT-scan (computed tomography) or a combination of these. The main aim of the study was twofold: to evaluate the outcomes of RCC surveillance to get insight in the safety of annual US and to evaluate the compliance to RCC surveillance.

## Materials and methods

Data of 199 BHD patients diagnosed with BHD at the VU University Medical Center and The Netherlands Cancer Institute were collected. Data were collected from the first screening until June 2014. The collection of family data and the methods of mutation analysis have been outlined in previous publications [3, 12]. Mutation testing and family counselling took place from 2004, after discovery of the *FLCN* gene. In all 199 patients, the diagnosis was confirmed by *FLCN* mutation testing. Our cohort includes both symptomatic index patients and healthy family members identified after pre-symptomatic DNA testing. Screening data and data on RCC were retrieved from the local medical files. In addition, the researchers sent a questionnaire on surveillance performed in other medical institutions to all patients in 2014. The patients were asked for the dates of screening, the imaging modality and findings and they were asked to give consent to collect these medical data. Sixty-six patients returned the questionnaire. The Medical Ethics Review Committee of the VUmc confirmed that the Medical Research Involving Human Subjects Acts (WMO) did not apply to this study. Official approval of the study was therefore not required. Patient data were anonymized.

For evaluation of compliance with surveillance, we assessed initial screening and follow-up screening data. Initial screening was defined as the first renal imaging performed in a patient after being diagnosed with BHD. For all patients who had initial screening, this took place within maximum one year after the genetic diagnosis of BHD. Patients diagnosed with (symptomatic) RCC before the diagnosis of BHD was established, were not considered to have had initial screening, but only follow-up screening. Follow-up was defined as all screening that took place after the initial screening or after the RCC diagnosis. Patients were included in the analysis of follow-up, if follow-up data until maximum 1.5 years before the end of the study (June 2014) or 1.5 years before death were complete.

## Results

### Screening compliance

**Fig 1:**
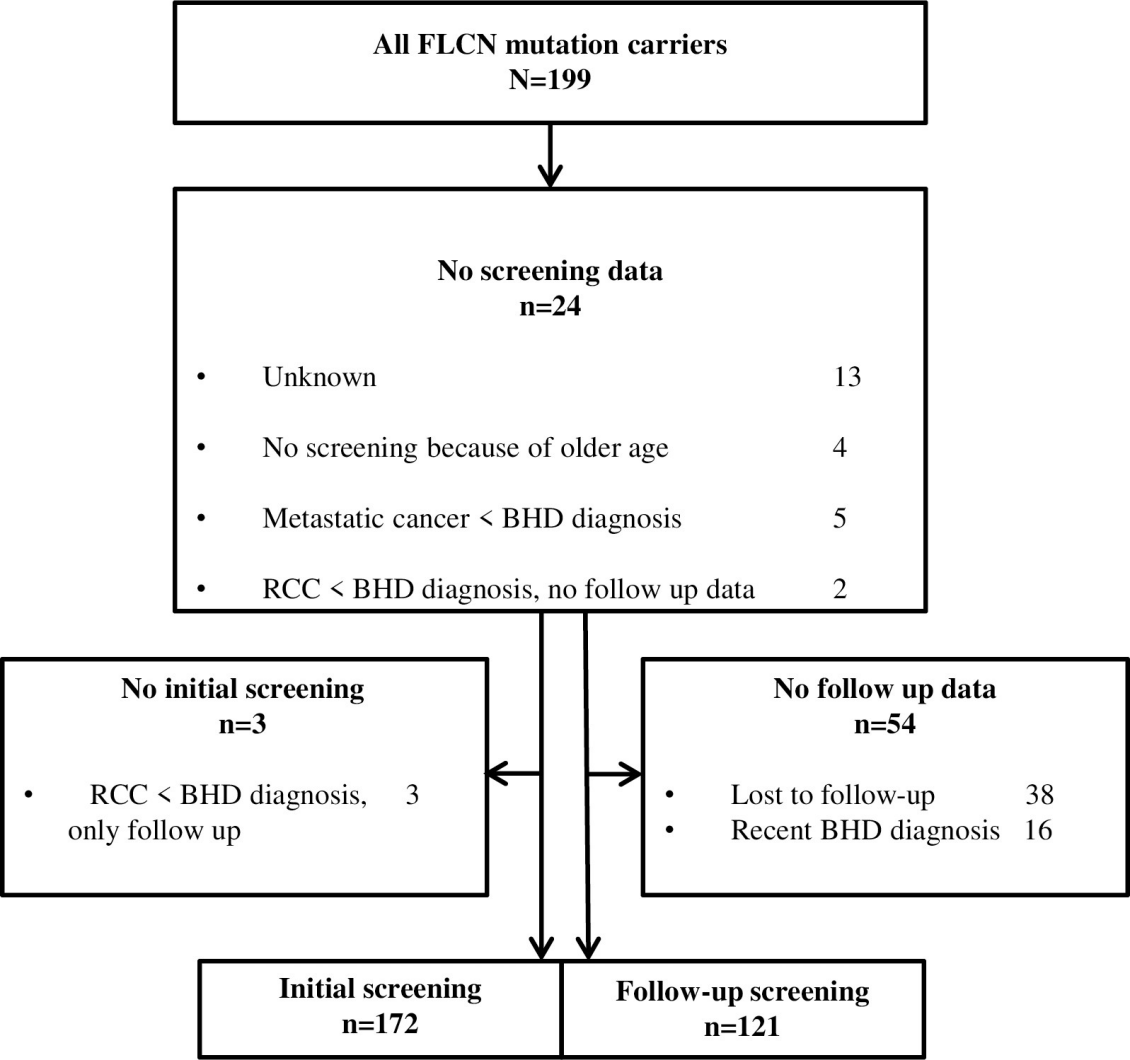
Screening data of 199 BHD patients.

Fig 1 shows a flowchart of the screening performed in the cohort. For 24 of 199 patients, we had no screening data at all. Some of these patients declined screening because of, for example, old age or having metastatic cancer. For 13 of these 24 patients, it was unknown whether screening was performed. They did not return the questionnaire and were not screened in one of the two hospitals participating in this study.

All patients with initial screening data (n = 172) underwent initial screening within 1 year after being diagnosed with BHD. Follow-up data could not be collected for 54 patients. In 16 of them, BHD had been diagnosed recently, so no follow-up was necessary yet. The other 38 patients did not return the questionnaire and were not screened in one of the two hospitals participating in this study. They might however undergo screening via their general practitioner in a regional hospital.

The mean age at initial screening was 50 (median 51, range 20–83). The data of initial and follow-up screening data are shown in Table 1. The mean follow-up period was 4.2 years (median 4; range 1–9). Most of the missed screening moments occurred in the first year of follow-up (13%). From the second to ninth year approximately 3–9% of patients per year missed a screening moment. The majority of patients (101/121, 83%) was screened at least once a year and missed no screening moment, 114/121 patients (94%) were screened at least once every two years, so they did not miss more than one successive screening moment in Table 1.

**Table 1:** Overview of number of patients that underwent initial and follow-up screening per year.

	Initial screening	Year 1	Year 2	Year 3	Year 4	Year 5	Year 6	Year 7	Year 8	Year 9
**Total number of patients in follow-up**	172	121	105	91	72	49	33	28	17	10
**MRI + US**	120 (69.8%)	18	17	14	12	11	7	5	2	0
**US**	9 (5.2%)	61	57	46	43	30	17	12	10	10
**MRI**	31(18.0%)	3	5	7	2	0	2	3	1	0
**Other**^**1**^	9 (5.2%)	4	1	3	1	1	0	1	1	0
**Technique unknown**^**2**^	3 (1.7%)	19	20	14	9	4	4	6	2	0
**No screening (%)**	NA	16 (13.2%)	5 (4.8%)	7 (7.7%)	5 (6.9%)	3 (6.1%)	3 (9.1%)	1 (3.6%)	1 (5.9%)	0 (0.0%)

^1^ CT, CT and US or CT and MRI

^2^ Self-reported screening with questionnaire in which technique was not mentioned.

NA: not applicable

### RCC

A total of 23 patients (13 male, 10 female) had been diagnosed with RCC. Table 2 shows tumour and patient characteristics. The mean age at diagnosis of the first RCC was 51 (range 24–77). In total, 38 tumours were detected in the 23 patients. Of one tumour no data was available since the treatment took place 34 years ago. Tthis tumour is not mentioned in the table. The histology was available for 29 tumours and was either chromophobe (44.8%), clearcell (24.1%), mixed chromophobe/clearcell (10.3%), papillary (6.9%), hybrid oncocytic/chromophobe (3.4%), sarcomatoid (3.4%), mixed chromophobe/clearcell/papillary (3.4%) or unclassified (3.4%). Histology was unavailable in the four untreated tumours, in three tumours treated with cryo-ablation or RFA, and the histology reports of one patient were unavailable due to missing consent.

**Table 2:** Detected RCCs: Tumour and patient characteristics.

Sex	Age at dx	Tumour size (mm)	Moment of dx	Symptoms	Detected with	Missed with	Last screening before dx	Duration of follow-up after last tumour
F	74	40 (PA)	<BHD	AS	CT	NA	NA	7 years FU
		60 (PA)	<BHD		CT			
		20 (PA)	<BHD		CT			
		11 (PA)	<BHD		CT			
M	51	69	<BHD	S	CT	NA	NA	Metastasis at diagnosis, died
M	56	120	<BHD	S	CT + US	NA	NA	Metastasis at diagnosis, died
M	56	51 (PA)	<BHD	AS	CT	NA	NA	Died due to metastasis of
		23 (PA)	<BHD		CT			other malignancy
		17 (PA)	<BHD		CT			
F	25	110 (PA)	<BHD	S	CT + US	NA	NA	21 years FU
M	42	14	<BHD	AS	CT	NA	NA	2 years FU
		8^1^	<BHD		CT			Current size unknown
M	69	55 (multifocal)	<BHD	AS	CT	NA	NA	Unknown
		15–20 (multifocal)	<BHD					
F	40	30	<BHD	U	MRI + US	NA	NA	10 years FU
M	24	70	<BHD	U	MRI+US	NA	NA	Metastasis at diagnosis, died
M	28	110	<BHD	U	MRI + US	NA	NA	Metastasis at diagnosis, died
F	55	27	Initial	AS	MRI	US (2 mo. earlier)	NA	3 years FU
M	69	3^1^	Initial	U	MRI	NA	NA	3 years (tumour is 9 mm after 3 years)
M	31	18	Initial	AS	MRI (+2nd US)	US (same day)	NA	3 years FU
F	53	19	Initial	S	CT	NA	NA	2 years FU
F	64	16	Initial	AS	MRI	US (6 weeks earlier)	NA	4 years FU
		7^1^	Initial		MRI	US (6 weeks earlier)	NA	Current size unknown
		5^1^ (6 mo. later)	Initial		MRI	NA	NA	Current size unknown
M	76	15	Initial	AS	CT	US (3 weeks earlier)	NA	4 years FU
		20	Initial		CT + US	NA		
		22	Initial		CT	US (3 weeks earlier)		
M	43	10	Initial	AS	MRI + CT	NA	6 mo bef: US	NA
		14	Initial			NA		
F	49	17	Initial	AS	MRI + CT	NA	NA	NA
M	33	90	Initial	U	MRI +US	NA	NA	2 years FU (after last tumour)
	35	13	FU		MRI	US (same day)	1 yr. bef: MRI+US	
	39	30	FU		MRI	NA	6 mo. bef: US	
F	77	11	FU	AS	CT	US (3 weeks earlier)	6 mo. bef: US	1 year FU
M	29	22	FU	U	MRI + US	NA	1 yr. bef: MRI+US	NA
F	60	50	FU	U	US	NA	Unknown	5 years FU
F	62	14	FU	U	MRI	US (interval unknown)	1 yr. bef: MRI+US	NA

^1^ Tumour was not treated and is in follow-up.

Mo.: months; yr.: years; dx: diagnosis. Sex: F; female, M; male. Tumour size: reported as size on imaging when available. In case of both US and CT/MRI is size on CT or MRI reported. In case of both CT and MRI, the largest described size is reported. PA; size measured by pathologist, no imaging size available. Moment of diagnosis: < BHD; diagnosis of RCC made before the diagnosis of BHD, FU; tumour diagnosed at follow-up, initial; tumour diagnosed at initial screening. Symptoms: U; unknown, AS; asymptomatic, S: symptomatic. Outcome: NA; not applicable

An ultrasound (US) was performed in 18 of the 38 tumours around the time of diagnosis. Nine tumours, sized 20 to 120 mm, were detected by US. Nine tumours, sized 7 to 27 mm, were seen on MRI or CT but not seen with US. The histology of the tumours not seen by US was either chromophobe (n = 4), clearcell (n = 1), mixed chromophobe/clearcell (n = 1) or unknown (n = 3, due to RFA treatment or no data). Four patients died from RCC, they all were diagnosed with metastatic before the diagnosis of BHD. One patient died from metastasis of a malignancy unrelated to BHD. No patients were diagnosed with metastasized RCC during follow-up.

## Discussion

To gain insight in optimal screening regimens in rare genetic cancer predisposition syndromes, it is crucial to study available patient cohort data. We aimed to collect data from 199 Dutch patients diagnosed with BHD in two Dutch centers. The recommended screening program in the Netherlands has changed over the years and may vary between hospitals. Initial screening in BHD patients often consisted of both MRI and US followed by annual US. Currently, initial screening usually consists of MRI only. The goal of initial screening by both MRI and US was to gain more insight in the optimal screening technique, since there are no evidence based guidelines for screening in BHD patients. The change to MRI only was based on expert opinion. As expected, the data show that the majority of performed initial screening consisted of both MRI and US (70%) and that the majority (53%) of follow-up screening consisted of US only.

In some patients annual imaging was performed in one of the two hospitals participating in this study. However, the majority of patients were screened in local or regional hospitals. This makes it difficult to remain up to date on the screening compliance and outcomes. Using questionnaires, we tried to collect these data of as many patients as possible. The presented data show that at least 86% (172/199) of the BHD patients underwent initial screening. Excluding patients with a diagnosis of RCC before the diagnosis of BHD, patients with metastatic cancer before the diagnosis of BHD and patients who underwent no screening because of old age, this increases to 93% (172/185). At least 61% (121/199) of the BHD patients performed follow-up screening. Excluding patients who underwent no screening because of old age, patients with metastatic cancer before the diagnosis of BHD and patients with a recent diagnosis of BHD, this increases to 70% (121/174).

Our cohort of patients with BHD participating in the screening program (n = 121) is highly compliant to the recommended screening regimen (83%). The fact that the most missed screening moments were in the first year after initial screening, might be due to less awareness of the patients own responsibility for organizing follow-up screening via their general practitioner. This might be improved by addressing this during genetic counseling or at initial screening. The exact number of patients declining screening and their reasons to do so remain unclear. The screening compliance is comparable to that reported in patients with Lynch syndrome (87%) [13].

The biological behaviour of RCC in BHD is reported to be indolent and metastases rarely occur at smaller tumour sizes. Taking into account the relatively high frequency of multiple or bilateral tumours, parenchymal sparing surgical treatment is important in patients with a hereditary predisposition for renal carcinoma [14, 15]. The aims of the treatment are local tumour control, preservation of renal function and prevention of metastatic disease. Nephron sparing treatment is often possible, since these slow growing tumours are often discovered at a small size. The ‘3 cm rule’, which recommends surgical intervention when the (largest) lesion exceeds 3 cm in diameter, is often applied to patients with Von Hippel Lindau disease, hereditary papillary RCC and BHD [2, 14, 16, 17]. In a previous study of 49 patients with hereditary RCC, no metastatic disease was reported in more than 10 years follow-up when adhering to the ‘3 cm rule’. It must be noted that in this study only 1 patient with BHD was included [15]. In our group of 23 BHD patients diagnosed with RCC, most patients had local treatment. The treatment of tumours smaller than 3 cm was possibly performed at the request of the patient due to anxiety. In addition, the increasing availability of radiofrequency ablation and cryotherapy for small tumours may have played a role in these decisions. When screening BHD patients, it is crucial that no tumours larger than 3 cm are missed by the applied screening technique and the screening interval must ensure that no tumours larger than 3 cm develop during the screening interval.

The main techniques to be considered for the imaging of renal tumours are CT, MRI and US. These imaging modalities each have their strengths and weaknesses. CT is a fast and robust technique to display the kidneys three-dimensionally and with great anatomical detail. CT is the gold standard for the diagnosis and staging of renal cell carcinoma [18]. Despite these qualities, the use of CT for annual surveillance might lead to unacceptably high cumulative radiation doses as patients would require many CT scans during their lifetime [19]. MRI is a technique that provides excellent soft tissue contrast without the use of radiation. It is powerful for the detection and characterization of focal renal lesions, various RCC subtypes can be differentiated and patency of blood vessels can be visualised without the use of intravenous contrast. Drawbacks of MRI are long examination times (30–45 minutes), variations in quality and scan protocols, limited availability and high costs [17]. In some patients MRI cannot be performed due to claustrophobia, obesity, or the presence of metallic implants. US has the advantages of being widely available, fast, cheap and lacking ionizing radiation. However, small renal lesions can be missed due to limited spatial resolution of US, depending on intrarenal location, isoechoic aspect of a lesion, obesity or obscuring bowel gas [14, 17, 20]. Furthermore US sensitivity depends on operator experience. Renal imaging with 1–3 year intervals has been proposed for BHD patients without renal lesions on initial imaging [11, 14, 21].

Using US, 9 of 18 tumours were missed in our cohort. All missed tumours were smaller than 3 cm (largest 27 mm). The histology of the missed tumours was comparable to that of the detectable tumours, suggesting that the histology is no major factor in the chance of missing a tumour on US. Consistent with current literature, no metastatic disease occurred in patients with RCC smaller than 3 cm. Our findings further emphasize the need for early diagnosis of BHD and performing screening from age 20 onwards or from the moment BHD is diagnosed. All 4 patients with metastatic RCC in our cohort, developed this disease before being aware of having BHD and two of them were only in their twenties. Since most RCCs were diagnosed at initial screening and BHD related RCCs are reported to have an indolent nature, it is likely that part of the tumours in our cohort could have been detected at an earlier time, if screening would have been performed.

Based on the findings in our cohort, annual US may be a safe and less expensive alternative for MRI, since only tumours smaller than 3 cm were missed on US. However, there are multiple disadvantages of this approach. First, early detection of a renal mass is likely to motivate patients to undergo repeated follow-up screening whereas false negative outcomes might lead to complacency. Second, if patients are not compliant to the annual surveillance, a small tumour might grow beyond 3 cm in the interval between imaging. Third, a tumour just under 3 cm might be missed with US and if it happens to grow relatively fast, might grow beyond 3 cm within a year. The final choice for MRI and US might also depend on local factors. The costs for individual patients and for the healthcare system of MRI and US might differ between countries. In the current Dutch situation, annual MRI is a far more expensive for the patient than annual US. The high costs of MRI might discourage patients from undergoing repeated screening. The final decision on the optimal surveillance should be made based on the above mentioned limitations and might differ between countries and even between patients. A compromise between the choice for MRI or US, might be annual surveillance with alternating MRI and US.

## Conclusions

Our main findings are that initial screening was performed in the vast majority of patients and that 85% of patients in the screened cohort was compliant with annual renal imaging. Ultrasound might be a sensitive, cheap and widely available alternative for MRI or part of the MRIs for detecting renal lesions larger than 3 cm in BHD patients. However, US has limitations and all of them should be carefully considered when determining the optimal surveillance. It might be important to give more attention to the recommended screening program, especially to the first follow-up moment after initial screening. Further studies are needed to determine whether our results for BHD patients in this study can be reproduced, preferably in a relatively large group of patients screened both by MRI and by ultrasound.
